# Routine data in a primary care performance dashboard, Ethiopia

**DOI:** 10.2471/BLT.23.291122

**Published:** 2024-05-07

**Authors:** Catherine Arsenault, Anagaw Derseh Mebratie, Solomon Kassahun Gelaw, Dessalegn Shamebo

**Affiliations:** a Department of Global Health, Milken Institute School of Public Health, The George Washington University, 950 New Hampshire Ave, NW Washington, DC 20052, United States of America.; b Department of Health Systems Management and Health Policy, Addis Ababa University School of Public Health, Addis Ababa, Ethiopia.; c Strategic Affairs Executive Office, Ministry of Health, Addis Ababa, Ethiopia.; d Department of Development Economics, Ethiopian Civil Service University, Addis Ababa, Ethiopia.

## Abstract

**Objective:**

To explore the feasibility of building a primary care performance dashboard using DHIS2 data from Ethiopia’s largest urban (Addis Ababa), agrarian (Oromia) and pastoral (Somali) regions.

**Methods:**

We extracted 26 data elements reported by 12 062 health facilities to DHIS2 for the period 1 July 2022 to 30 June 2023. Focusing on indicators of effectiveness, safety and user experience, we built 14 indicators of primary care performance covering reproductive, maternal and child health, human immunodeficiency virus, tuberculosis, noncommunicable disease care and antibiotic prescription. We assessed data completeness by calculating the proportion of facilities reporting each month, and examined the presence of extreme outliers and assessed external validity.

**Findings:**

At the regional level, average completeness across all data elements was highest in Addis Ababa (82.9%), followed by Oromia (66.2%) and Somali (52.6%). Private clinics across regions had low completeness, ranging from 38.6% in Somali to 58.7% in Addis Ababa. We found only a few outliers (334 of 816 578 observations) and noted that external validity was high for 11 of 14 indicators of primary care performance. However, the 12-month antiretroviral treatment retention rate and proportions of patients with controlled diabetes or hypertension exhibited poor external validity.

**Conclusion:**

The Ethiopian DHIS2 contains information for measuring primary care performance, using simple analytical methods, at national and regional levels and by facility type. Despite remaining data quality issues, the health management information system is an important data source for generating health system performance assessment measures on a national scale.

## Résumé

**Objectif:**

Déterminer s'il est possible de créer un tableau de bord des performances de soins de santé primaires en utilisant les données du DHIS2 issues des plus grandes régions urbaines (Addis-Abeba), agricoles (Oromia) et pastorales (Somali) d'Éthiopie.

**Méthodes:**

Nous avons extrait 26 éléments de données transmis au DHIS2 par 12 062 établissements de santé pour la période allant du 1^er^ juillet 2022 au 30 juin 2023. En nous focalisant sur l'efficacité, la sécurité et l'expérience vécue par les patients, nous avons défini 14 indicateurs de performances pour les soins de santé primaires, couvrant des domaines tels que la santé reproductive, maternelle et infantile, le virus de l'immunodéficience humaine, la tuberculose, la prise en charge des maladies non transmissibles et la prescription d'antibiotiques. Nous avons évalué l'exhaustivité des données en calculant le pourcentage d'établissements produisant un rapport mensuel, avant d'examiner la présence de valeurs aberrantes extrêmes et la validité externe.

**Résultats:**

À l'échelle régionale, c'est à Addis-Abeba (82,9 %) que l'exhaustivité moyenne pour l'ensemble des éléments de données était la plus élevée, suivie d'Oromia (66,2 %) et de Somali (52,6 %). Dans toutes les régions, les cliniques privées affichaient un faible taux d'exhaustivité compris entre 38,6 % au Somali et 58,7 % à Addis-Abeba. Nous n'avons trouvé que quelques valeurs aberrantes (334 sur 816 578 observations) et avons constaté un haut degré de validité externe pour 11 des 14 indicateurs de performances des soins de santé primaires. Toutefois, le degré de validité du taux de maintien du traitement antirétroviral sur 12 mois ainsi que de la proportion de patients avec une hypertension ou un diabète sous contrôle s'est révélé moindre.

**Conclusion:**

Le DHIS2 éthiopien contient des informations utiles pour mesurer les performances des soins de santé primaires à l'aide de méthodes analytiques simples, tant au niveau national que régional et selon les types d'établissements. Malgré les quelques problèmes qui subsistent en termes de qualité, ce dispositif de gestion des informations sanitaires représente une précieuse source de données pour élaborer des outils permettant d'évaluer les performances du système de santé à l'échelle nationale.

## Resumen

**Objetivo:**

Explorar la viabilidad de crear un tablero de rendimiento de la atención primaria utilizando datos DHIS2 de las principales regiones urbanas (Addis Abeba), agrarias (Oromia) y pastorales (Somali) de Etiopía.

**Métodos:**

Se extrajeron 26 elementos de datos notificados por 12 062 centros sanitarios a DHIS2 para el periodo comprendido entre el 1 de julio de 2022 y el 30 de junio de 2023. A partir de indicadores de eficacia, seguridad y experiencia de los usuarios, se elaboraron 14 indicadores del rendimiento de la atención primaria que abarcan la salud reproductiva, materna e infantil, el virus de la inmunodeficiencia humana, la tuberculosis, la atención de enfermedades no transmisibles y la prescripción de antibióticos. Se evaluó la exhaustividad de los datos al calcular el porcentaje de centros que informaban cada mes y se examinó la presencia de valores atípicos y de la validez externa.

**Resultados:**

A nivel regional, el nivel medio de exhaustividad de todos los datos fue más alto en Addis Abeba (82,9%), seguida de Oromia (66,2%) y Somalia (52,6%). Las clínicas privadas de todas las regiones presentaban un bajo nivel de exhaustividad, que oscilaba entre el 38,6% en Somalia y el 58,7% en Addis Abeba. Solo se encontraron unos pocos valores atípicos (334 de 816 578 observaciones) y se observó que la validez externa era alta para 11 de los 14 indicadores de rendimiento de la atención primaria. Sin embargo, la tasa de retención del tratamiento antirretroviral a los 12 meses y los porcentajes de pacientes con diabetes o hipertensión controladas mostraron una escasa validez externa.

**Conclusión:**

El DHIS2 etíope contiene información para medir el rendimiento de la atención primaria, utilizando métodos analíticos sencillos, a escala nacional y regional y por tipo de centro. A pesar de los problemas de calidad de los datos que aún persisten, el sistema de información sobre la gestión sanitaria es una fuente de datos importante para generar medidas de evaluación del rendimiento del sistema sanitario a escala nacional.

## ملخص

**الغرض:**

استكشاف جدوى تأسيس لوحة معلومات لأداء الرعاية الأولية باستخدام بيانات DHIS2، من أكبر المناطق الحضرية (أديس أبابا)، والزراعية (أوروميا)، والرعوية (صومالي) في إثيوبيا.

**الطريقة:**

قمنا باستخراج 26 عنصرًا للبيانات من التي أبلغت عنها 12062 منشأة صحية لـ DHIS2، للفترة من 1 يوليو/تموز 2022 إلى 30 يونيو/حزيران 2023. وبالتركيز على مؤشرات الفعالية والسلامة وتجربة المستخدم، قمنا بوضع 14 مؤشرًا لأداء الرعاية الأولية تغطي كل من الصحة الإنجابية، والأمومة، وصحة الطفل، وفيروس نقص المناعة البشرية، والسل، ورعاية الأمراض غير المعدية، ووصف المضادات الحيوية. قمنا بتقييم مدى اكتمال البيانات من خلال حساب نسبة المنشآت التي تقدم تقاريرها كل شهر، وفحصنا وجود القيم المتطرفة، والصلاحية الخارجية.

**النتائج:**

على المستوى الإقليمي، كان متوسط الاكتمال عبر كل عناصر البيانات الأعلى في أديس أبابا (%82.9)، وتليها أوروميا (%66.2)، ثم صومالي (%52.6). وبالنسبة لمدى اكتمال العيادات الخاصة عبر المناطق، فقد كانت منخفضة، حيث تراوحت من %38.6 في صومالي، إلى %58.7 في أديس أبابا. لقد عثرنا على عدد قليل فقط من القيم المتطرفة (334 من 816578 من الملاحظات)، ولاحظنا أن الصلاحية الخارجية كانت مرتفعة لعدد 11 من 14 مؤشرًا لأداء الرعاية الأولية. ومع ذلك، فإن معدل المداومة على العلاج المضاد للفيروسات القهقرية لمدة 12 شهرًا، ونسب المرضى الذين يعانون من مرض السكري أو ارتفاع ضغط الدم، الخاضع للسيطرة، فقد أظهروا صلاحية خارجية ضعيفة.

**الاستنتاج:**

يحتوي نظام DHIS2 الإثيوبي على معلومات لقياس أداء الرعاية الأولية، باستخدام طرق تحليلية بسيطة، على الصعيدين الوطني والإقليمي، وحسب نوع المنشأة. وعلى الرغم من المشكلات المتبقية المتعلقة بجودة البيانات، فإن نظام معلومات الإدارة الصحية يعد مصدرًا هامًا للبيانات لوضع مقاييس التقييم لأداء النظام الصحي على المستوى الوطني.

## 摘要

**目的:**

探讨利用埃塞俄比亚最大城市(亚的斯亚贝巴)、农业区(奥罗米亚)和牧区(索马里)的 DHIS2 数据构建初级保健绩效仪表板的可行性。

**方法:**

我们提取了在 2022 年 7 月 1 日至 2023 年 6 月 30 日期间,12,062 家卫生机构向 DHIS2 报告的 26 种数据元素。以有效性、安全性和用户体验指标为重点,我们制定了涵盖生殖、母婴健康、人体免疫缺陷病毒、结核病、非传染性疾病护理和抗生素处方的 14 个初级保健绩效指标。我们通过计算每月进行报告的卫生机构比例评估了数据完整性,同时还研究了极端异常值的情况和外部有效性。

**结果:**

在区域层面,亚的斯亚贝巴所有数据元素的平均完整性最高 (82.9%),其次是奥罗米亚 (66.2%) 和索马里 (52.6%)。各区域私人诊所的完整性较低,介于 38.6%(索马里)和 58.7%(亚的斯亚贝巴)之间不等。我们仅发现了少数异常值(816,578 份观察结果中仅有 334 份存在异常),同时发现,在 14 项初级保健绩效指标中,共有 11 项的外部有效性较高。但是,在接受抗逆转录病毒治疗 12 个月后的药物留存率以及糖尿病或高血压患者病情控制率方面,显示外部有效性较低。

**结论:**

通过利用埃塞俄比亚 DHIS2 系统提供的信息,我们仅需使用简单分析方法即可在国家和区域层面按卫生机构类型衡量初级保健绩效。尽管仍存在数据质量问题,但卫生管理信息系统始终是在全国范围内制定卫生系统绩效评估措施的重要数据来源**。**

## Резюме

**Цель:**

Изучить возможность создания панели мониторинга эффективности оказания первичной медико-санитарной помощи с использованием данных DHIS2 из крупнейших городских (Аддис-Абеба), аграрных (Оромия) и пастушеских (Сомали) регионов Эфиопии.

**Методы:**

В период с 1 июля 2022 года по 30 июня 2023 года было получено 26 элементов данных, представленных 12 062 медицинскими учреждениями в DHIS2. Сосредоточив внимание на показателях эффективности, безопасности и удобства использования, авторы разработали 14 показателей оценки эффективности оказания первичной медико-санитарной помощи, охватывающих репродуктивное здоровье, здоровье матери и ребенка, вирус иммунодефицита человека, туберкулез, помощь при неинфекционных заболеваниях и назначение антибиотиков. Для оценки полноты данных рассчитывалась доля учреждений, представивших отчеты за каждый месяц, а также проверялось наличие экстремальных значений и внешняя обоснованность.

**Результаты:**

На региональном уровне средний показатель полноты данных по всем элементам был самым высоким в Аддис-Абебе (82,9%), за ним следуют Оромия (66,2%) и Сомали (52,6%). Частные клиники в разных регионах отличались низкой полнотой охвата: от 38,6% в Сомали до 58,7% в Аддис-Абебе. Было обнаружено лишь несколько отклонений (334 из 816 578 наблюдений), и отмечается, что внешняя обоснованность была высокой для 11 из 14 показателей оценки эффективности первичной медико-санитарной помощи. Однако показатели продолжительности 12-месячного антиретровирусного лечения и доля пациентов с контролируемым сахарным диабетом или артериальной гипертензией были недостаточно достоверными с точки зрения внешней обоснованности**.**

**Вывод:**

В ПО DHIS2 Эфиопии представлена информация для оценки эффективности первичной медико-санитарной помощи с помощью простых аналитических методов на национальном и региональном уровнях и по типам учреждений. Несмотря на сохраняющиеся проблемы с качеством данных, информационная система управления здравоохранением является важным источником данных для разработки показателей оценки эффективности системы здравоохранения в национальном масштабе.

## Introduction

Functioning primary care is essential for improving population health. A greater emphasis on primary care in the health-care system can reduce costs, improve access and reduce inequities in population health. In Ethiopia, a country with great unmet health-care needs, the performance and quality of the primary health-care system are suboptimal.^1^^–^^3^ The coronavirus disease 2019 (COVID-19) pandemic, as well as the ongoing armed conflict in the northern part of the country, have further threatened the resilience of the health-care system.^4^^–^^6^

Evidence on health system performance in Ethiopia has been limited by the availability and type of data used for assessment. Most studies have relied on data collected through household or health facility surveys, methods which are costly and infrequent.^7^^,^^8^

The government-owned health system is structured into three tiers: primary facilities such as health centres and, in rural areas, health posts and primary hospitals; secondary facilities (general hospitals); and tertiary facilities (specialized hospitals). The private health sector is present at all levels of care, ranging from primary care facilities to private for-profit hospitals and specialty centres.^9^ Outpatient primary care is delivered across all levels of the health system but it remains unclear whether primary care performance varies according to the facility type where it is provided. In particular, the performance of the Ethiopian private sector has received only limited attention in literature.

The capacity to measure and use data for learning and improvement is a foundation of a high-quality health system.^2^ DHIS2 (formerly known as the District Health Information System 2) is an open-source, web-based health management information system platform designed to routinely generate health statistics to support decision-making. DHIS2 is a widely used platform, over 80 countries have adopted it, and DHIS2 supports routine health management for an estimated 3.2 billion people.^10^ Motivated by a need to improve data use for health system improvement, the Ethiopian health ministry adopted DHIS2 in 2016 as part of its digital health plan, achieving national implementation in 2019. The use of DHIS2 offers various benefits, including global support, standardization and flexibility. The system’s open-source nature allows customization to meet each country’s specific needs. The Ethiopian DHIS2 contains regularly submitted data, typically monthly, by all 30 192 health facilities in the country. The system includes reports on health service activities and the conditions for which people seek care.^11^

Concerns about data quality have hampered use of DHIS2. As a result, its rapid and extensive scaling up has not been matched by a corresponding increase in data use by policy-makers and researchers.^12^ DHIS2 data remain under-represented in the scientific literature.^13^ The Ethiopian health ministry conducts annual reviews of health system performance using DHIS2 data.^14^ However, the majority of indicators monitored focus on coverage estimates (e.g. antenatal care coverage or immunization coverage) that do not reflect the quality of the services provided and rely on denominators that are sometimes inaccurate (e.g. estimated number of pregnant women, expected number of infants).

Beyond coverage measures, it remains unclear whether DHIS2 data can be used to track health system performance and to compare performance by facility type. In this paper, we explore the feasibility of building a primary care performance dashboard using DHIS2 data in three regions of Ethiopia. We conduct a detailed assessment of DHIS2 data quality by region and facility type, and discuss the availability of indicators to assess primary care performance.

## Methods

Our analysis includes all health facilities reporting at least one of the indicators of interest to DHIS2 in three regions of Ethiopia: the largest urban (Addis Ababa), agrarian (Oromia) and pastoral (Somali) regions in the country. We extracted the monthly data at the health facility level. We categorized the facilities as: health posts (Oromia and Somali); health centres, private clinics, public hospitals and private hospitals (Addis Ababa and Oromia). Ethiopia uses its own 13-month calendar (the *Ge’ez* calendar). However, for budgeting and planning purposes the government uses a 12-month fiscal year that corresponds to 1 July to 30 June of the Gregorian calendar. For this analysis, we extracted data for Ethiopian Fiscal Year 2015, which corresponds to 1 July 2022 to 30 June 2023, using the pivot table module in DHIS2. We exported the data as csv files for analysis using Stata version 18 (Stata Corp. LP, College Station, United States of America).

To select sentinel measures of primary care performance, we first reviewed all data elements included in the Ethiopian DHIS2. Following the health system performance assessment framework for universal health coverage, we selected indicators of primary care effectiveness, safety and user experience.^15^ We aimed to include indicators that covered a range of primary care needs for different health conditions, including reproductive, maternal and child health indicators, human immunodeficiency virus (HIV), tuberculosis and noncommunicable disease care. We included measures of timely care (e.g. proportion of pregnant women who had their first antenatal care visit in the first trimester); appropriate care (e.g. proportion of pregnant women tested for syphilis and HIV during antenatal care); treatment effectiveness (tuberculosis treatment success rate, viral load suppression in patients living with HIV, blood pressure and blood sugar control); and retention in care (proportion of women attending four antenatal care visits of those having at least one visit; proportion of children receiving the third dose of pentavalent vaccine of those receiving the first dose; or proportion of children receiving the second dose of rotavirus vaccine of those receiving the first dose; and proportion of patients living with HIV still on antiretroviral therapy [ART] 12 months after initiation). The proportion of patients receiving an antibiotic was also included as a measure of treatment effectiveness, but also relates to patient safety and antimicrobial resistance.^16^ Care retention reflects the user’s experience and their willingness to continue receiving care.

We assessed four dimensions of data quality: reporting completeness, presence of outliers, internal consistency and external validity. For each data element, we assessed reporting completeness over the year by calculating the proportion of facilities reporting each month relative to the total number of facilities reporting at least once during the year. We also checked each data element for the presence of extreme positive outliers. We defined outliers as any observation greater than three standard deviations from the facility-level mean over the year, among volumes that were greater than 100 clients.^17^ We set any outlier found to be missing before describing the results. The statistical code used for the data quality assessment and removal of outliers is publicly available in an online repository.^18^

We assessed internal consistency by building the performance indicators (i.e. dividing one data element by the other) and ensuring that numerators did not surpass denominators at the regional and facility-type levels. To assess external validity, we triangulated the regional-level performance indicators with estimates from external sources, including the 2016 Ethiopia Demographic and Health Survey (DHS);^19^ the 2019 Ethiopia Mini DHS;^7^ the Ministry of Health Ethiopian Fiscal Year 2015 performance report;^14^ the 2021–2022 Ethiopia Service Provision Assessment survey;^8^ and estimates from World Health Organization (WHO) and Joint United Nations Programme on HIV/AIDS (UNAIDS).^19^^–^^22^ The primary care performance dashboard was built using the data elements aggregated annually for the Ethiopian fiscal year 2015. The primary care performance indicators were also disaggregated by region and facility type.

The Institutional Review Board of The George Washington University determined that this study is not human subjects research, and exempted the study from a full review.

## Results

From DHIS2, we extracted a total of 26 data elements, which we used to calculate 14 primary care performance indicators (Box 1). A total of 15 578 facilities were listed in DHIS2 across the three regions. However, 3516 did not report any of the 26 data elements needed for analysis during the year. Our analytical data set therefore included 12 062 health facilities and 144 744 facility-month observations.

Box 1 **Data elements extracted from DHIS2 and primary care performance indicators calculated, Ethiopia, July 2022 to June 2023**

**Data elements**

(i) total no. of immediate postpartum contraceptive acceptors; (ii) total no. of births attended by skilled health personnel; (iii) no. of women attended first antenatal care visit; (iv) no. of first antenatal care visits in the first trimester;(v) no. of pregnant women attended four antenatal care visits; (vi) no. of pregnant women tested for syphilis; (vii) no. of pregnant women tested for HIV; (viii) no. of pregnant women who received iron and folic acid; (ix) no. of children with first dose of pentavalent vaccine; (x) no. of children with third dose of pentavalent vaccine; (xi) no. of children with first dose of rotavirus; (xii) no. of children with second dose of rotavirus; (xiii) no. of people still on ART 12 months after initiation; (xiv) no. of people initiated on ART; (xv) no. of ART patients with an undetectable viral load (< 50 copies/mL); (xvi) no. of ART patients for whom a viral load test was done at 12 months; (xvii) no. of tuberculosis patients cured; (xviii) total no. of tuberculosis patients on treatment; (xix) no. of hypertensive patients with controlled blood pressure at 6 months; (xx) no. of hypertensive patients enrolled in care 6 months prior; (xxi) no. of diabetic patients with controlled blood sugar at 6 months; (xxii) no. of diabetic patients enrolled in care 6 months prior; (xxiii) no. of patient encounters with one or more antibiotics; (xxiv) total no. of patient encounters at facilities; (xxv) total outpatient visits; and (xxvi) total number of new and repeat acceptor of oral contraceptives.

**Performance indicators**

Reproductive health: proportion of women accepting immediate postpartum contraceptive (counselling effectiveness)

Antenatal care: proportion of pregnant women receiving timely antenatal care (timely care); proportion attended four antenatal care visits (care continuity); syphilis testing coverage; HIV testing coverage; and iron and folic acid provision (appropriate care)

Routine immunization: retention to the third pentavalent vaccine dose; and retention to the second rotavirus vaccine dose (care continuity)

HIV: proportion of people living with HIV on ART after 12 months (care continuity); and % of people with a viral load suppression (treatment effectiveness)

Tuberculosis: treatment success rate (treatment effectiveness)

Hypertension: proportion of patients with controlled blood pressure (treatment effectiveness)

Diabetes: proportion of patients with controlled blood sugar (treatment effectiveness)

Antibiotic prescribing: Proportion of patients receiving antibiotics (appropriate care, safety)

Reporting completeness for each data element and region is shown in Table 1 (available at: https://www.who.int/publications/journals/bulletin/). At the regional level, average completeness across all data elements was highest in Addis Ababa (82.9%), followed by Oromia (66.2%) and Somali (52.6%). Completeness was above 70% for all data elements in Addis Ababa except for tuberculosis. Previously, tuberculosis data elements were reported in Ethiopia on a quarterly basis. Some facilities may therefore be lagging in transitioning their reporting practices to a monthly frequency. In Oromia and Somali, seven and 11 data elements had completeness less than 50%, respectively. Facility deliveries and childhood vaccination had the highest reporting completeness (more than 75% in all three regions). Diabetes, hypertension and tuberculosis data elements had the lowest reporting completeness. Overall, we found few extreme outliers, less than 0.1% of observations (334/816 578).

**Table 1:** Average reporting completeness of data elements, by region, Ethiopia, July 2022 to June 2023

Data element	Monthly average, %		
	Addis Ababa	Oromia	Somali
**Total outpatient visits**	73.6	77.9	70.2
**Reproductive health**			
Oral contraceptives	80.2	84.0	58.7
Postpartum contraceptives	76.3	49.1	27.4
Facility deliveries	93.7	93.0	78.7
**Antenatal care**			
First visit	92.6	67.4	67.4
First visit in first trimester	83.9	60.3	48.4
Four visits	89.2	67.8	66.3
Syphilis testing	92.1	80.6	61.9
HIV testing	90.2	79.2	46.8
Iron and folic acid provision	88.5	69.5	52.1
**Routine immunization**			
Pentavalent vaccine dose 1	93.9	89.3	75.3
Pentavalent vaccine dose 3	93.3	89.4	75.3
Rotavirus vaccine dose 1	92.4	88.5	75.4
Rotavirus vaccine dose 2	93.1	88.6	75.0
**HIV**			
ART at 12 months	73.9	58.5	51.7
Initiation of ART	77.0	64.4	60.8
Viral load undetected	86.7	59.7	43.8
Viral load tested	86.1	63.3	46.3
**Tuberculosis**			
Patients cured	63.7	49.0	30.7
Total no. of patients	63.7	49.6	30.8
**Hypertension**			
Controlled blood pressure	79.9	47.5	34.8
No. of patients enrolled	80.7	47.9	35.6
**Diabetes**			
Controlled blood sugar	70.3	37.1	25.3
No. of patients enrolled	75.3	37.2	26.4
**Antibiotic prescribing**			
At least one antibiotic	82.4	61.5	50.8
Total patient encounters	82.2	62.1	50.7

ART: antiretroviral therapy; HIV: human immunodeficiency virus.

Note: Data element definitions are provided in Box 1. We obtained the data from the Ethiopian DHIS2. Completeness is defined as the number of facilities reporting each month divided by the total number of facilities that reported each data element at least once during the year. The table shows average completeness over 12 months.

Table 2 presents the primary care performance dashboard at the regional level as well as internal and external validity assessments. Only one indicator had poor internal consistency at the regional level, where the number of women receiving iron and folic acid during pregnancy in Oromia was slightly higher than the total number of first antenatal care visits, suggesting that iron and folic acid are delivered outside of antenatal care visits. Eleven out of 14 indicators demonstrated strong external validity, as their estimates closely aligned with those obtained from external sources. Three indicators had poor external validity: 12-month ART retention rate, and proportions of patients with controlled diabetes or hypertension. The 12-month ART retention rate ranged from only 27% to 50% across the three regions. However, a systematic review of 45 studies from Ethiopia, with varying lengths of follow-up, found an average ART retention rate of 70.7%;^23^ and a study in 22 sub-Saharan African countries also found the ART retention rate at year 1 to be 76.8%.^24^ Our estimate from DHIS2 may be affected by unaccounted losses to follow-up or by poor reporting completeness. The low retention rate also does not coincide with the relatively high rate of viral suppression. The proportions for diabetes and hypertension control also had poor external validity and were substantially higher than expected, at 77.6% and 77.4% on average, respectively (Table 2) compared with 34.4% and 37.5%, according to a systematic review and the 2023 WHO Global Report on Hypertension.^22^^,^^25^ DHIS2 reporting for hypertension and diabetes is fairly recent in Ethiopia, and the numbers of patients enrolled in care may be poorly captured.

**Table 2:** A primary care performance dashboard in three regions of Ethiopia, July 2022 to June 2023

Data element or indicator	Addis Ababa (405 facilities)^a^	Oromia (10 102 facilities)^a^	Somali (1 555 facilities)^a^	Average	External comparison
**Reproductive health**					
Women accepting immediate postpartum contraception, no.	23 295	141 120	3 367	NA	NA
Births attended by skilled health personnel, no.	153 626	1 107 606	82 554	NA	NA
Immediate postpartum contraceptive acceptance, %	15.2	12.7	4.1	10.7	8.0^14^
**Antenatal care**					
Pregnant women attending first antenatal care visit, no.	190 916	1 506 219	203 938	NA	NA
First antenatal care visits in the first trimester, no.	44 957	332 985	39 404	NA	NA
Women receiving timely antenatal care, %	23.5	22.1	19.3	21.7	22.0–37.7^7^^,^^14^
Pregnant women attending four antenatal care visits, no.	166 583	1 062 412	129 593	NA	NA
Women retained to fourth antenatal care visit, %	87.3	70.5	63.5	73.8	58.0–79.0^7^^,^^14^
Pregnant women tested for syphilis, no.	187 518	1 202 867	95 392	NA	NA
Syphilis testing coverage, %	98.2	79.9	46.8	75.0	65.0–74.0^8^^,^^14^
Pregnant women tested for HIV, no.	173 853	1 070 449	49 095	NA	NA
HIV testing coverage, %	91.1	71.1	24.1	62.1	59.0^19^
Pregnant women receiving iron and folic acid, no.	152 684	1 710 910	145 813	NA	NA
Iron and folic acid provision, %	80.0	113.6^b^	71.5	88.4	67.0–77.0^7^^,^^14^
**Routine immunization**					
Children with first dose of pentavalent vaccine, no.	144 678	1 546 208	203 460	NA	NA
Children with third dose of pentavalent vaccine, no.	142 194	1 466 519	180 964	NA	NA
Children receiving third pentavalent vaccine dose, %	98.3	94.8	88.9	94.0	80.3^7^
Children with first dose of rotavirus vaccine, no.	139 129	1 512 186	199 329	NA	NA
Children with second dose of rotavirus vaccine, no.	138 926	1 429 770	179 552	NA	NA
Children receiving second rotavirus vaccine dose, %	99.9	94.5	90.1	94.8	92.0^7^
**HIV **					
People still on ART 12 months after initiation, no.	6 188	43 140	537	NA	NA
People initiated on ART, no.	12 369	92 922	1 999	NA	NA
People on ART after 12 months^c^, %	50.0	46.4	26.9	41.1	70.7–76.8^23^^,^^24^
ART patients with an undetectable viral load (< 50 copies/mL), no.	84 321	278 635	985	NA	NA
ART patients for whom a viral load test was done at 12 months, no.	91 895	311 931	1 203	NA	NA
People with a viral load of suppression, %	91.8	89.3	81.9	87.7	81.0–96.4^14^^,^^21^
**Tuberculosis**					
Tuberculosis patients cured, no.	2 648	19 216	1 168	NA	NA
Tuberculosis patients on treatment, no.	2 829	19 834	1 326	NA	NA
Tuberculosis treatment success, %	93.6	96.9	88.1	92.9	86.0^20^
**Hypertension**					
Hypertensive patients with controlled blood pressure at 6 months, no.	24 942	244 374	5 217	NA	NA
Hypertensive patients enrolled in care 6 months prior, no.	34 914	303 406	6 507	NA	NA
Patients with hypertension control^c^, %	71.4	80.5	80.2	77.4	37.5^22^
**Diabetes**					
Diabetic patients with controlled blood sugar at 6 months, no.	15 078	83 226	2 638	NA	NA
Diabetic patients enrolled in care 6 months prior, no.	21 132	103 163	3 264	NA	NA
Patients with diabetes control^c^, %	71.4	80.7	80.8	77.6	34.4^25^
**Antibiotics**					
Patient consultations with antibiotic prescriptions, no.	1 700 554	11 931 264	351 440	NA	NA
Patient consultations, no.	4 094 391	18 595 369	677 852	NA	NA
Patient consultations resulting in antibiotic prescription, %	41.5	64.2	51.8	52.5	60.0^26^

ART: antiretroviral therapy; HIV: human immunodeficiency virus; NA: not applicable.

^a^ Number of facilities reporting varies for each data element.

^b^ Number of prescriptions for iron and folic acid was higher than number of antenatal care visits in Oromia region.

^c^ Indicators had poor external validity and results are likely inaccurate.

Notes: We obtained the data from the Ethiopian DHIS2. Inconsistencies arise in some values due to rounding.

**Fig. 1:**
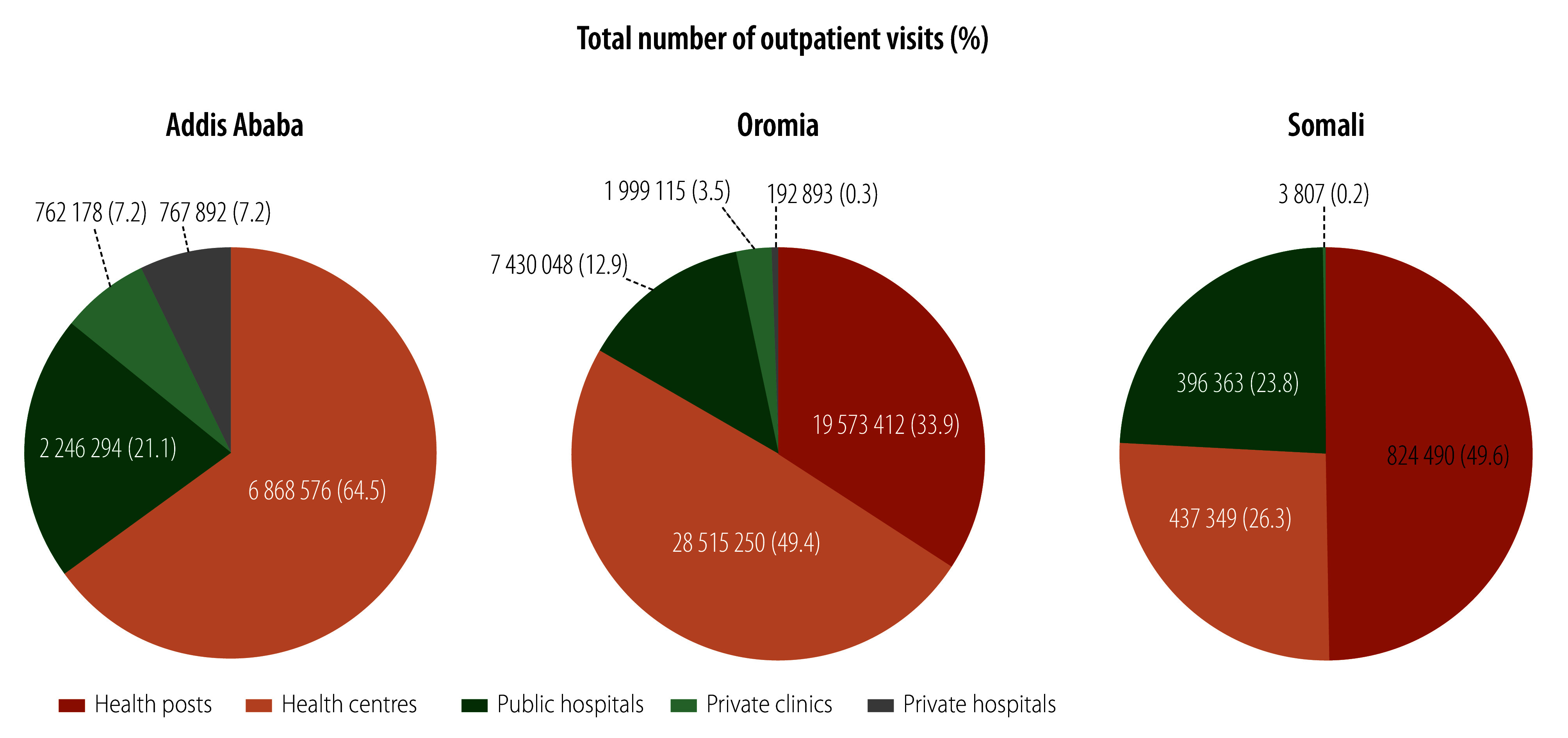
Primary care service volume by facility type in three regions of Ethiopia, July 2022 to June 2023

Note: Data reported to the DHIS2 system over the year. Inconsistencies arise in some values due to rounding.

**Fig. 2:**
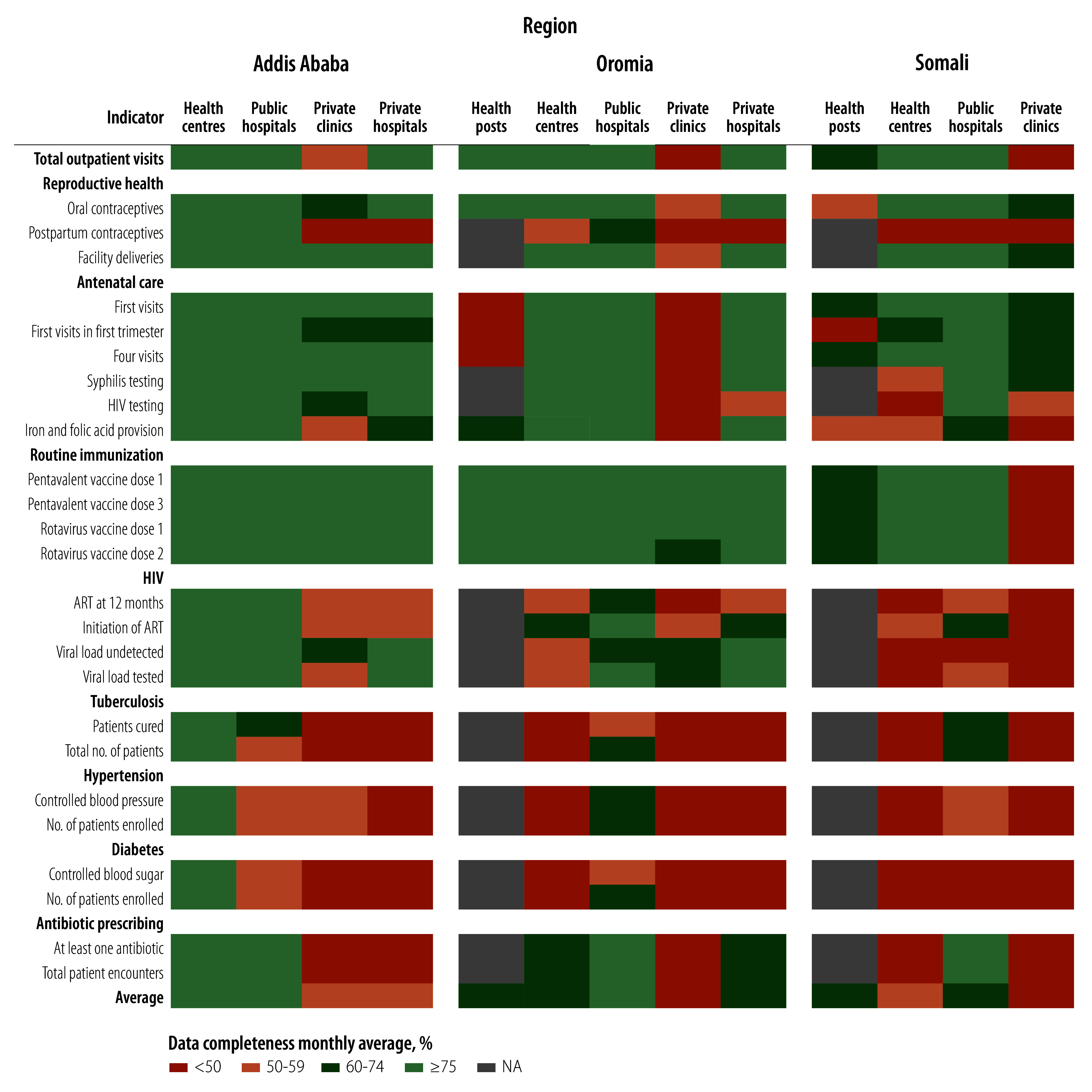
Average reporting completeness of data elements, by facility type, Ethiopia, July 2022 to June 2023

ART: antiretroviral therapy; HIV: human immunodeficiency virus; NA: not applicable.

Note: Data elements definitions are provided in Box 1. Number of facilities per type and region is given in Table 3. Data elements marked as NA are not reported by health posts. We obtained the data from the Ethiopian DHIS2. Completeness is defined as the number of facilities reporting each month divided by the total number of facilities that reported each data element at least once during the year. The table shows average completeness over 12 months.

When assessing the proportion of total outpatient visits reported by each facility type, 15.8% of all visits across all three regions were reported by hospitals. Private sector primary care was low (less than 3.8%) in both Oromia and Somali, but accounted for 14.4% of reported primary care in Addis Ababa (Fig. 1). We assessed reporting completeness and internal consistency by region and facility type (Fig. 2). Completeness across all data elements was highest in Addis Ababa health centres (92.9% on average), followed by public hospitals in Addis Ababa and Oromia. In contrast, completeness was low in private clinics in all regions, ranging from 38.6% in Somali to 58.7% in Addis Ababa (Fig. 2 and online repository).^18^ The primary care performance indicators showed interesting trends by facility type (Table 3). For example, syphilis and HIV testing during antenatal care was not higher in hospitals compared with health centres, despite the former being generally better equipped. The 2021–2022 Service Provision Assessment survey also found that antenatal syphilis testing was equivalent in health centres (76%) compared with hospitals (75%).^8^ Furthermore, in Addis Ababa the ART retention rate was high (67.0%) in health centres, where reporting accuracy is notably high, compared with reported rates in other facility types (about 40.0%; Table 3). In the disaggregated analyses, two indicators had poor internal consistency. First, public hospitals in Addis Ababa saw twice as many women in their fourth or subsequent antenatal care visit compared with women in their first antenatal care visit (Table 3). This outcome is probably because some women were referred to public hospitals from the primary level for follow-up antenatal care. The primary care performance indicators by facility type may be biased if patients move to different levels of the health system throughout the year. Second, in health posts in Oromia, provision of iron and folic acid was six times the number of antenatal care visits, indicating that iron and folic acid are delivered outside of these visits

**Table 3:** Primary care performance indicators by region and facility type, Ethiopia July 2022 to June 2023

Data element or indicator	Addis Ababa					Oromia						Somali			
	Health centres (97)	Public hospitals (12)	Private clinics (272)	Private hospitals (24)		Health posts (6 682)	Health centres (1 398)	Public hospitals (108)	Private clinics (1 904)	Private hospitals (9)		Health posts (1 314)	Health centres (218)	Public hospitals (18)	Private clinics (5)
**Reproductive health**															
Women accepting immediate postpartum contraception, no.	14 311	7 217	1 713	54		NA	117 012	17 012	2 632	1 051		NA	2 064	306	4
Births attended by skilled health personnel, no.	55 884	64 858	22 953	9 931		NA	876 622	213 838	12 864	4 282		NA	56 521	22 951	3 082
Immediate postpartum contraceptive acceptance, %	25.6	11.1	7.5	0.5		NA	13.3	8.0	20.5	24.5		NA	3.7	1.3	0.1
**Antenatal care**															
Pregnant women attending first antenatal care visit, no.	131 318	12 351	36 001	11 246		112 931	1 143 497	178 445	65 109	6 237		83 255	77 726	37 275	5 682
First antenatal care visits in the first trimester, no.	33 140	1 715	7 496	2 606		14 641	283 568	20 309	13 346	1 121		17 052	14 893	6 234	1 225
Women receiving timely antenatal care, %	25.2	13.9	20.8	23.2		13.0	24.8	11.4	20.5	18.0		20.5	19.2	16.7	21.6
Pregnant women attending four antenatal care visits, no.	105 165	25 712	27 975	7 731		75 001	851 089	89 855	40 980	5 487		58 718	56 316	9 371	5 188
Women retained to fourth antenatal care visit, %	80.1	208.2	77.7	68.7		66.4	74.4	50.4	62.9	88.0		70.5	72.5	25.1	91.3
Pregnant women tested for syphilis, no.	130 997	9 305	36 651	10 565		NA	998 201	149 173	51 340	4 153		NA	55 239	33 933	6 220
Syphilis testing coverage, %	99.8	75.3	101.8	93.9		NA	87.3	83.6	78.9	66.6		NA	71.1	91.0	109.5
Pregnant women tested for HIV, no.	129 171	6 276	28 451	9 955		NA	924 660	124 831	18 075	2 883		NA	18 591	26 114	4 390
HIV testing coverage, %	98.4	50.8	79.0	88.5		NA	80.9	70.0	27.8	46.2		NA	23.9	70.1	77.3
Pregnant women receiving iron and folic acid, no.	127 992	6 715	11 927	6 050		679 890	908 058	120 270	0	2 692		76 946	55 452	13 415	0
Iron and folic acid provision, %	97.5	54.4	33.1	53.8		602.0	79.4	67.4	0.0	43.2		92.4	71.3	36.0	0.0
**Routine immunization**															
Children with first dose of pentavalent vaccine, no.	116 301	1 474	20 263	6 640		1 036 570	443 092	53 000	13 474	72		111 628	72 153	17 676	2 003
Children with third dose of pentavalent vaccine, no.	117 499	1 318	17 308	6 069		996 363	413 576	43 973	12 541	66		99 411	65 484	14 392	1 677
Children receiving third pentavalent vaccine dose, %	101.0	89.4	85.4	91.4		96.1	93.3	83.0	93.1	91.7		89.1	90.8	81.4	83.7
Children with first dose of rotavirus vaccine, no.	111 966	1 378	19 450	6 335		1 015 717	433 562	49 716	13 117	74		108 964	70 939	17 489	1 937
Children with second dose of rotavirus vaccine, no.	114 418	1 322	17 549	5 637		967 613	405 647	44 060	12 380	70		98 259	65 123	14 588	1 582
Children receiving second rotavirus vaccine dose, %	102.2	95.9	90.2	89.0		95.3	93.6	88.6	94.4	94.6		90.2	91.8	83.4	81.7
**HIV **															
People still on ART 12 months after initiation, no.	3 205	820	1 511	652		NA	35 156	7 466	444	74		NA	14	523	NA
People initiated on ART, no.	4 786	2 037	3 723	1 823		NA	74 187	17 881	747	115		NA	323	1 676	NA
People on ART after 12 months^c^, %	67.0	40.3	40.6	35.8		NA	47.4	41.8	59.4	64.3		NA	4.3	31.2	NA
ART patients with an undetectable viral load (< 50 copies/mL), no.	41 790	28 351	7 450	6 730		NA	119 897	145 567	6 837	6 334		NA	33	952	NA
ART patients for whom a viral load test was done at 12 months, no.	45 027	30 917	8 178	7 773		NA	135 584	162 507	7 548	6 292		NA	53	1 150	NA
People with a viral load of suppression, %	92.8	91.7	91.1	86.6		NA	88.4	89.6	90.6	100.7		NA	62.3	82.8	NA
**Tuberculosis**															
Tuberculosis patients cured, no.	2 286	89	170	103		NA	16 663	2 289	206	58		NA	411	713	44
Tuberculosis patients on treatment, no.	2 418	96	207	108		NA	17 131	2 421	222	60		NA	486	796	44
Tuberculosis treatment success, %	94.5	92.7	82.1	95.4		NA	97.3	94.5	92.8	96.7		NA	84.6	89.6	100.0
**Hypertension**															
Hypertensive patients with controlled blood pressure at 6 months, no.	18 214	5 200	1 020	508		NA	169 322	67 752	6 680	620		NA	2 091	3 102	24
Hypertensive patients enrolled in care 6 months prior, no.	24 041	8 830	1 261	782		NA	205 383	88 839	8 410	774		NA	2 680	3 803	24
Patients with hypertension control^c^, %	75.8	58.9	80.9	65.0		NA	82.4	76.3	79.4	80.1		NA	78.0	81.6	100.0
**Diabetes**															
Diabetic patients with controlled blood sugar at 6 months, no.	8 012	5 663	403	1 000		NA	25 389	52 787	4 243	807		NA	479	2 141	18
Diabetic patients enrolled in care 6 months prior, no.	11 220	8 066	528	1 318		NA	29 523	67 706	5 039	895		NA	720	2 526	18
Patients with diabetes control^c^, %	71.4	70.2	76.3	75.9		NA	86.0	78.0	84.2	90.2		NA	66.5	84.8	100.0
**Antibiotics**															
Patient consultations with antibiotic prescriptions, no.	1 350 937	297 953	42 361	9 303		NA	9 200 967	2 591 411	99 978	38 908		NA	85 924	264 864	652
Patient consultations, no.	2 983 719	1 028 499	57 846	24 327		NA	12 832 068	5 594 030	120 005	49 266		NA	108 352	567 085	2 415
Patient consultations resulting in antibiotic prescription, %	45.3	29.0	73.2	38.2		NA	71.7	46.3	83.3	79.0		NA	79.3	46.7	27.0

ART: antiretroviral therapy; HIV: human immunodeficiency virus; NA: not applicable.

^a^ Indicators had poor internal consistency with values >  100%.

^b^ Indicators had poor external validity.

Notes: Indicator definitions are provided in Box 1. We obtained the data from the Ethiopian DHIS2. Indicators marked as NA are not available in health posts.

## Discussion

In this paper, we have explored the feasibility of building a primary care performance dashboard using DHIS2 data from 12 062 health facilities in Ethiopia, corresponding to 40.0% of the 30 192 health facilities in the country. A total of 26 data elements were used to build 14 primary care performance indicators, including indicators of timely care, appropriate treatment, treatment effectiveness, safety and patient retention, which are important dimensions of service quality and intermediate objectives of health systems.^15^

Our data quality assessment revealed that reporting completeness is low in the Somali region compared with Addis Ababa and Oromia. Somali is one of four regions in Ethiopia where the predominant occupation is pastoralism. Furthermore, this region also has one of the weakest infrastructures in the country, characterized by a scarcity of health facilities and a shortage of health-care providers.^8^^,^^27^

Reporting completeness was also low in private facilities. Therefore, our estimate of the proportion of primary care provided by the private sector may be underestimated. The performance indicators in private facilities may also be biased downward if numerators had poorer completeness than denominators. Although we included all facilities reporting to DHIS2 over the year, some active facilities, particularly in the private sector, may still be missing from DHIS2. A Master Facility Registry, listing all existing facilities in Ethiopia, is being developed but is not currently integrated with DHIS2.^14^ Therefore, we are unsure of the true number of private facilities operating in the country. Poor reporting in the private sector has been described in other countries, and will require targeted approaches to incentivize private facilities to improve their reporting practices.^28^^,^^29^ Nonetheless, our findings on reporting completeness must be interpreted with caution. In DHIS2, low completeness may indicate true missing data, where facilities are failing to report, but can also mean that the facilities did not have any patients for a certain service in a particular month. Zero counts are not reported in DHIS2 and appear as missing in the data set, an important limitation of the platform that has been raised by others.^30^ Some facilities may also aggregate data over several months and only report once (e.g. per quarter) if the internet is not available in a given month, for example. Since we aggregated results over 12 months in our dashboard, only true missing data, where facilities are failing to report despite having patients, would bias the results.

Our findings also reveal that although most primary care services were delivered in public health centres or health posts, between 13.2% (7 622 941/57 718 718) and 28.3% (3 014 186/10 644 940) of total outpatient visits took place in hospitals. The provision of primary care in hospitals is not always recommended due to gaps in continuity, poorer user experience and higher costs.^31^ Studies have shown that an increasing number of people in low- and middle-income countries are opting for hospitals or private sector facilities to receive primary care.^31^^–^^35^

DHIS2 data offer important advantages and opportunities for improving the assessment of health system performance in low- and middle-income countries. First, unlike population- or facility-based surveys conducted only every 4–5 years, DHIS2 data are reported monthly by all health facilities in the country, allowing frequent assessments on a national scale.^19^ For example, during the COVID-19 pandemic, many researchers turned to DHIS2 data to generate timely evidence on the magnitude of disruptions to health services.^4^^,^^5^^,^^10^^,^^12^^,^^36^^,^^37^ The use of one health management information system across all facility types and regions also facilitates standardized comparisons on a national scale. Moreover, unlike surveys, conducting performance assessments through DHIS2 does not require extra investments in data collection beyond the existing maintenance costs. Finally, DHIS2 data are locally led and government owned, decreasing reliance on international bodies for health system performance assessment.

Nonetheless, DHIS2 data continue to face limitations, including poor reporting completeness for some indicators, facilities or regions. This drawback will require improvements in data quality and reporting at the point of data collection. Upon reviewing all available data elements, we also observed certain ambiguities in indicator definitions. This issue has been documented in other countries as well.^30^ Careful documentation of definitions and guidelines for reporting is crucial to improve data quality. To standardize reporting, WHO has collaborated with the health information systems programme at the University of Oslo, Norway, responsible for DHIS2, to create toolkits for specific programme areas (e.g. HIV and immunization) that include DHIS2 configuration packages.^38^ WHO should also include health system performance measures in these toolkits. In Ethiopia, DHIS2 data are also currently limited to facility-level aggregates. Incorporating patient-level information through electronic health records, for example, would allow more precise measures of care quality. Other countries have begun to incorporate individual-level data in DHIS2, including through the DHIS2 tracker module, which allows individual-based data processing and follow-up of people under different programmes, such as antenatal care, ART or routine immunizations. Concerning the availability of indicators, we found a large number pertaining to maternal and child health, whereas fewer were dedicated to noncommunicable disease care. Additionally, the Ethiopian DHIS2 lacks any indicator pertaining to mental health. Most data elements included in DHIS2 also aim to track service use and patient counts rather than service performance and quality of care. Finally, given the complexity and size of DHIS2 data sets, improving data use will also require the building of local data science skills.

We have shown that the DHIS2 system in Ethiopia contains important measures of primary care performance and that, despite some data limitations, 11 of the indicators presented had good external validity. Previously, DHIS2 has been primarily used to estimate health intervention coverage, such as proportion of deliveries conducted in facilities or the proportion of children vaccinated. These estimates were often limited by unreliable denominators estimating the size of catchment populations. The indicators we present in this paper do not rely on these denominators. The primary care performance dashboard should be repeated on an annual basis across all regions to monitor changes in primary care performance. The primary care performance indicators could also be estimated at zonal or district levels for a more granular assessment of performance. Assuming similar indicators are available in the DHIS2 systems of other countries, the dashboard could be replicated elsewhere.^30^

Despite important investments in scaling up the DHIS2 system in recent years, this expansion has not been matched by a corresponding increase in information use.^12^ Efforts to improve DHIS2 data demand and data use require improvements in DHIS2 data quality.^39^ Governments and researchers should harness DHIS2 more effectively to generate performance assessment measures that are valuable for policy-making and improvement. The analyses presented here aim to contribute to this effort by providing a new framework to monitor primary care performance using DHIS2.
